# Dietary Advanced Glycation End Products and Risk Factors for Chronic Disease: A Systematic Review of Randomised Controlled Trials

**DOI:** 10.3390/nu8030125

**Published:** 2016-03-01

**Authors:** Rachel E. Clarke, Aimee L. Dordevic, Sih Min Tan, Lisa Ryan, Melinda T. Coughlan

**Affiliations:** 1 Glycation, Nutrition and Metabolism Laboratory, Baker IDI Heart and Diabetes Institute, Melbourne 3000, Australia; min.tan@bakeridi.edu.au (S.M.T.); melinda.coughlan@bakeridi.edu.au (M.T.C.); 2 Department of Nutrition and Dietetics, Monash University, Notting Hill 3168, Australia; aimee.dordevic@monash.edu (A.L.D.); lisa.ryan@gmit.ie (L.R.); 3 Department of Natural Sciences, Galway-Mayo Institute of Technology, Galway H91 T8NW, Ireland

**Keywords:** systematic review, advanced glycation end-products, diet, chronic kidney disease, diabetes, cardiovascular disease, inflammation

## Abstract

Dietary advanced glycation end-products (AGEs) form during heating and processing of food products and are widely prevalent in the modern Western diet. Recent systematic reviews indicate that consumption of dietary AGEs may promote inflammation, oxidative stress and insulin resistance. Experimental evidence indicates that dietary AGEs may also induce renal damage, however, this outcome has not been considered in previous systematic reviews. The purpose of this review was to examine the effect of consumption of a high AGE diet on biomarkers of chronic disease, including chronic kidney disease (CKD), in human randomized controlled trials (RCTs). Six databases (SCOPUS, CINHAL, EMBASE, Medline, Biological abstracts and Web of Science) were searched for randomised controlled dietary trials that compared high AGE intake to low AGE intake in adults with and without obesity, diabetes or CKD. Twelve dietary AGE interventions were identified with a total of 293 participants. A high AGE diet increased circulating tumour necrosis factor-alpha and AGEs in all populations. A high AGE diet increased 8-isoprostanes in healthy adults, and vascular cell adhesion molecule-1 (VCAM-1) in patients with diabetes. Markers of CKD were not widely assessed. The evidence presented indicates that a high AGE diet may contribute to risk factors associated with chronic disease, such as inflammation and oxidative stress, however, due to a lack of high quality randomised trials, more research is required.

## 1. Introduction

Lifestyle factors, such as diets high in fat, sugar and salt, play a key role in the development and progression of chronic diseases, such as type 2 diabetes (T2DM), cardiovascular disease (CVD) and chronic kidney disease (CKD) [1]. The modern Western diet is comprised of highly processed foods that are rich not only in fat, sugar and salt but also contain potentially pathogenic compounds known as advanced glycation end-products (AGEs). Two recent systematic reviews that examined the effect of dietary AGE consumption suggest a positive relationship between AGE intake and serum AGE levels, markers of inflammation, oxidative stress and insulin resistance [2,3]. However these reviews made conclusions based on animal, cohort, and cross-sectional studies rather than examining the true effect of dietary-AGEs on chronic disease markers through RCTs. Furthermore, biomarkers of renal function were not considered and as circulating AGEs are thought to be particularly toxic to the kidney [4,5,6,7,8] it is of interest whether a high AGE diet may contribute to the development and progression of CKD, a major co-morbidity of other chronic diseases.

Current World Health Organisation dietary recommendations for the prevention of chronic disease recommend limiting the intake of free sugars, saturated fat and salt [9]. At present there are no recommendations surrounding the consumption of foods high in AGEs, such as heat-treated milk or cereals [10], which may be perceived as contributing to a healthy diet. In order to guide dietary recommendations for dietetic practice it is important to determine whether high AGE diets impact the development and progression of chronic conditions. Therefore, the objective of this systematic review was to determine the effect of high dietary AGE intake compared to a low AGE intake on biomarkers of systemic inflammation, oxidative stress and other risk factors for T2DM, CVD and CKD. Secondary outcomes of circulating and excreted AGE levels were also considered in order to examine associations between dietary AGE consumption, absorption and metabolism.

## 2. Materials and Methods

### 2.1. Eligibility Criteria

This systematic review was conducted in accordance with the preferred reporting items for systematic review and meta-analyses protocols (PRISMA-P) 2015 statement [11]. Studies were limited to randomised controlled trials (RCTs) [12] and performed in human adults (>18 years of age) in healthy or overweight populations, or in populations with obesity, type 1 diabetes (T1DM), T2DM or CKD. Interventions were included if they involved the consumption of a diet high in AGE content, Maillard reaction products (MRP) or Amadori compounds (precursors to AGE formation) compared with an isocaloric low AGE/MRP diet. A high AGE diet was defined as containing at least 30% more measured AGEs, MRP or Amadori compounds than the comparator or cooked using a method known to increase the formation of AGEs. Studies that investigated the AGE content of parenteral feeds were excluded as the AGEs bypass the gastrointestinal tract. Studies that only considered the postprandial response to single meals of varying AGE content were also excluded from this review as adverse consequences of dietary AGE consumption are thought to occur over longer time frames; a minimum time-frame of 1 week was required for inclusion. Other eligibility criteria included publication in English language, having undergone peer review and considering at least one of the outcomes of interest. Publications from any date were considered. The PICO (Patient/Population; Intervention; Comparator; Outcome) question addressed in this review was as follows: in healthy and diseased adults (P), does a high AGE diet (I) compared to a low AGE diet (C), effect biomarkers of inflammation, oxidative stress and risk factors for chronic disease (O)?

### 2.2. Information Sources

In total six databases were searched (SCOPUS, CINHAL, EMBASE, Medline, Biological Abstracts and Web of Science) in April 2015. The Cochrane library of clinical trials was also searched. The following keywords were used: (Concept 1) (advanced glycation end product OR carboxymethyllysine OR pentosidine OR maillard reaction product OR dicarbonyl OR carboxyethyllysine OR amadori product OR crosslink OR pyrraline OR methylglyoxal) AND (Concept 2) (diet OR food OR intake OR exogenous OR nutrition OR western diet OR processed food OR eat OR consumption OR bakery product OR animal derived NEAR fat or protein OR canned food OR heat treated near food OR diet). No limits in terms of language or years were set on databases when searching. Subject headings were also searched in order to locate relevant studies that may not contain key words in the title or abstract. Reference lists of relevant original research and reviews articles were hand searched.

### 2.3. Selection Process

Titles and abstracts of publications returned from the search were screened for relevance by one reviewer. Selected articles were retrieved and assessed for inclusion using the criteria described. Studies which could not be clearly included or excluded were screened by a second reviewer.

### 2.4. Data Management and Collection

Endnote was used to store and manage references for all studies returned for each database. Microsoft Excel was used to collate extracted data. All data were extracted by using a predefined data extraction template that was based on the National Health and Medical Research Council (NHMRC) of Australia data extraction template for RCTs and cohort studies.

### 2.5. Data Items

Data extraction variables included: affiliation/source of funding; study design (crossover/parallel); study duration; location/setting; population size and characteristics including inclusion and exclusion criteria; dietary intervention details such as method of delivery, differences in AGE content, energy content and macronutrient composition; method of assessing AGE content of diet; method of randomisation; blinding; measurement and reporting of compliance to dietary intervention; medication and supplementation; and any additional risks of bias identified.

### 2.6. Outcomes and Prioritisation

The primary outcomes of interest extracted from each study were differences in: biomarkers of inflammation (tumour necrosis factor alpha (TNFα), interleukin-6 (IL-6), C reactive protein (CRP) and monocyte chemoattractant protein-1 (MCP-1)); oxidative stress (8-isoprostane); T2DM (homeostatic model assessment insulin resistance (HOMA IR), fasting blood glucose (FBG), haemoglobin A1_c_ (HbA1_c_); CVD (oxidised low density lipoprotein (OxLDL)), vascular cell adhesion molecule-1 (VCAM-1) and intracellular cell adhesion molecule-1 (ICAM-1)); and CKD (urine albumin, serum creatinine, estimated glomerular filtration rate (eGFR) and plasma Cystatin C). Secondary outcomes of interest were levels of circulating, urinary and faecal carboxymethyl lysine (CML) a well-characterised AGE.

### 2.7. Risk of Bias and Quality Assessment

The Cochrane risk of bias tool [13] was used to assess the likelihood of bias at the study level for each of the included studies. The key domains of interest were: adequate sequence generation (method of randomisation); adequate allocation concealment (whether researchers or participants could foresee assignment); blinding (participant blinding and outcome assessor blinding considered separately); whether incomplete data was addressed; free of selective outcome reporting; and free of any other bias (transparent reporting of dietary intake, inclusion and exclusion criteria of participants, any baseline differences between groups). The overall risk of bias for each study was determined based on the total score (high, low, or unclear risk of bias) under each of the described domains. In addition, the American Dietetic Association (ADA) quality criteria checklist [14] was used to assess the quality of the included studies. The quality assessment checklist provides an overall quality measure of positive, negative, or neutral based on: relevance of the study; clarity of research question; unbiased selection of participants; whether study groups were comparable; unbiased and transparent handling of withdrawals; whether blinding was used; quality of reporting of the intervention; whether outcomes were clearly defined and the measurement of outcomes was valid and reliable, including appropriate nutrition measures; appropriate use of statistics; inclusion of a discussion of limitations of the study; and whether any bias exists related to funding source or conflict of interest. Two reviewers independently performed the quality assessment and any disagreements were discussed until consensus was reached. 

### 2.8. Data Synthesis

Data for each of the primary and secondary outcomes were synthesised and where possible reported as means at baseline and end of the intervention for each study group. This data was used to calculate the effect size (Cohen’s *d*) in order to compare the results across the studies. A Cohen’s *d* of 0.2–0.3 was considered small; 0.5 medium; and 0.8 or greater as large. A negative sign indicates an adverse effect due to dietary intervention. For some studies it was not possible to calculate effect size due to pre and post-means or standard deviations not being reported.

## 3. Results

### 3.1. Studies Identified

**Figure 1:**
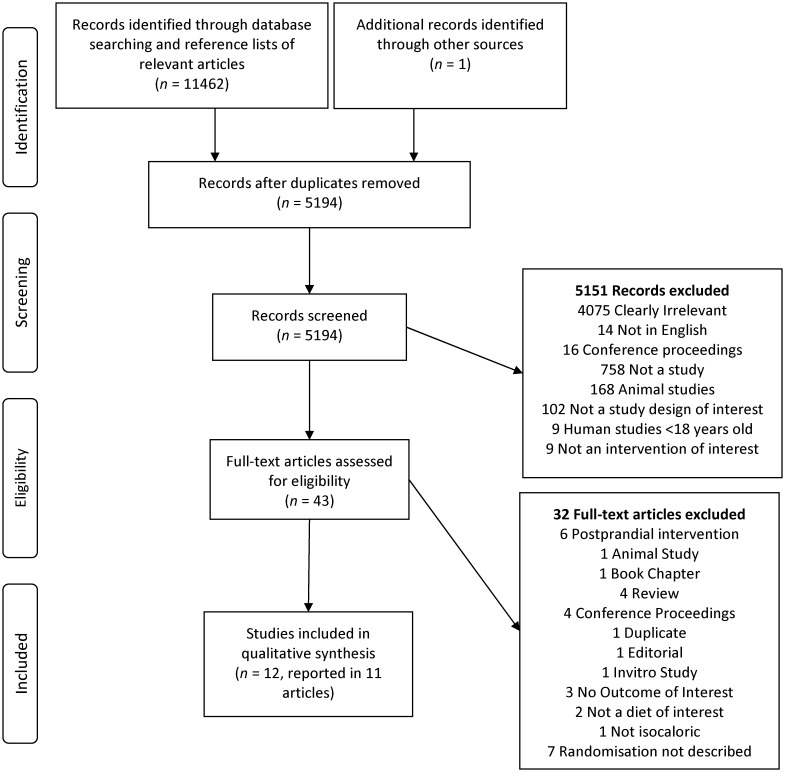
PRISMA Flow diagram of search results, screening and included studies.

A total of 5194 original articles were returned from the initial search (Figure 1). After screening of titles and abstracts, 43 full text articles were identified and retrieved for assessment. A total of 12 randomised controlled trials reported in 11 articles met eligibility criteria and were included in this review [15,16,17,18,19,20,21,22,23,24,25]. The total number of participants in the included studies was 293.

### 3.2. Study Characteristics

The characteristics of included studies are shown in Table 1. All studies used cooking methods to generate differences in AGEs between diets. In six cases the dietary intervention was a high AGE diet [15,16,18,20,24] with a low AGE diet as a comparator. In the remaining six studies a standard diet high in AGEs was compared to an AGE restricted diet [17,19,21,22,23,25]. In several studies the standard diets contained AGEs at levels similar to, or greater than, a high AGE diet used in another intervention, as measured by ELISA or estimated from an AGE database, and can therefore also be considered high AGE diets [15,19,22,23,24,25]. Participants were either provided study meals [15,16,18,20,24,25] or provided with instructions for meal preparation [17,19,21,22,23,25]. Dietary compliance was assessed by most studies using food diaries [16,17,19,21,23,24,25], a daily questionnaire [20], biweekly telephone calls [22], or a single telephone call over four-weeks [18]; however dietary compliance was not reported in one study [15]. Only one study [18] used liquid chromatography mass spectrometry (LC-MS) to measure the AGE content of the diets, three studies [15,24] used ELISA and eight studies [16,17,19,20,21,22,23,25] used the same reference database [26] to estimate AGEs in diets. Of the outcomes of interest considered in this review there were no studies that reported data for ICAM-1, eGFR, or faecal AGE content and only one study measured plasma cystatin C [16]. No studies reported any unexpected adverse outcomes due to the dietary interventions. 

### 3.3. Studies in Healthy Populations

#### 3.3.1. Biomarkers of Inflammation and Oxidative Stress

Three [21,22,25] studies in healthy individuals measured changes in circulating TNFα levels (Table 2). Of these, three [21,22,25] longer term studies (16-weeks) found that circulating TNFα was increased from baseline after consumption of the high AGE intervention compared with the low AGE intervention while the short-term study (two-weeks) did not observe differences between interventions [16]. The calculation of effect size [22] suggested a large negative effect on TNFα levels, or increase in this biomarker, due to the high AGE diet and a medium positive effect, or decrease, due to the low AGE diet [22]. 

Studies that measured circulating IL-6 [16,18] and CRP [16,20,21] levels following high AGE consumption from two to 16-weeks found no differences between interventions. Calculated effect sizes showed negligible effects on these biomarkers due to the high AGE diet [20] and a small but negative effect on CRP, indicating increased levels following the low AGE diet [20]. Only one study [16] measured MCP-1 and reported that there was an increase in this marker after only two-weeks of consumption of a high AGE diet compared with a low AGE diet in healthy overweight males. Three [21,22,25] studies reported increases in plasma levels of the oxidative stress marker 8-isoprostanes (a marker of lipid peroxidation) and one [16] observed an increase in urinary 8-isoprostanes after two-weeks consumption of a high AGE diet. Calculated effect size for one study [22] suggests the high AGE diet had a medium adverse effect on 8-isoprostanes levels, while the low AGE diet had a large positive effect in two studies [21,22]. 

#### 3.3.2. Biomarkers of Chronic Disease Risk

In healthy adults, a high AGE diet did not increase risk factors for T2DM including fasting blood glucose [16,18,20,22,25] or HbA1_c_ [22] (Table 3). One study reported an increase [18] in HOMA-IR after four-weeks on a high AGE diet while another long-term study observed no difference after 16-weeks [21] on a standard diet high in AGEs compared with a restricted AGE intake. The calculated effect sizes suggest a negligible or small effect due to the AGE content of the diet on T2DM risk factors including HbA1_c_ and HOMA-IR, and a negligible or small effect of a low or reduced AGE diet [18,20,22]. Three studies [20,21,25] measured VCAM-1; two studies where food was provided to participants reported no significant differences between dietary interventions [20,25], while the other [21], in which participants prepared their own foods, found higher circulating levels of VCAM-1. The effect size calculated for this study revealed no effect due to the high AGE diet and a small positive effect, or reduction in VCAM-1, attributed to a low AGE diet [20]. Markers of kidney disease in healthy adults were assessed within two studies [16,25]. One short-term study in healthy overweight males reported increased urinary albumin-to-creatinine ratio and increased plasma cystatin C after two-weeks on a high AGE diet [16], whereas neither study observed differences in serum creatinine or creatinine clearance, respectively [16,25].

#### 3.3.3. Circulating and Excreted CML

The effect of a high AGE diet on circulating CML varied with study duration (Table 4). Three long-term trials [21,22,25] in healthy adults reported an increase in circulating CML 16-weeks after consumption of a high AGE diet or standard diet high in AGEs compared with an AGE restricted diet. Calculated effect sizes indicate a large positive, or lowering, effect of a low AGE diet on serum AGEs across studies of varying durations (four to 16-weeks) [20,21,22]; the effect of a high AGE diet on serum AGEs is less clear with a small positive effect seen in one short-term study (four-weeks) and a medium negative, or increasing effect seen in a longer term study (16-weeks) [22]. A two-week crossover trial [16] in healthy overweight males found circulating AGEs reduced after the high AGE intervention compared with a restricted AGE diet whilst urinary AGE excretion increased. Only one other study [20] measured urinary CML in healthy adults and reported no differences between diets; similarly the calculated effect sizes were negligible due to both diets.

### 3.4. Studies in Patients with Diabetes

#### 3.4.1. Biomarkers of Inflammation and Oxidative Stress

Similar to the effects observed in healthy participants, studies in individuals with diabetes that measured TNFα found that the high AGE intervention resulted in significantly higher circulating levels [17,22,24] (Table 2). For one study it was possible to calculate the effect size [22] which demonstrated a large negative effect due to high AGE intervention and a medium positive effect of the low AGE diet on TNFα levels. Of the studies that measured CRP levels, two [17,24] reported no differences due to intervention while one [24] demonstrated that CRP increased after six-weeks on a high AGE diet. The only study [22] that measured 8-isoprostanes as a marker of oxidative stress in T2DM patients reported significantly higher mean plasma concentrations after 16-weeks on a standard diet high in AGEs. The effect size calculated for this study suggests that the adverse effect of the high AGE diet on 8-isoprostanes was moderate, while the low AGE diet was seen to have a large positive effect [22] (Table 2).

#### 3.4.2. Biomarkers of Chronic Disease Risk

The studies that examined the effect of a high AGE diet on insulin resistance in patients with diabetes were conflicting [17,22] (Table 3). The calculated effect size for one study suggested that the high AGE diet had a moderate negative effect on, or increased, HOMA-IR while the low-AGE diet had a large positive effect [22]. The majority of the included articles suggested that a high AGE diet has no effect on fasting blood glucose levels [15,17,22,24] or HbA1_c_ [22] in this population. CVD risk factors were reported to increase in patients with diabetes on a high AGE diet, though it was not possible to determine effect size for any study [15,24]. The one study that measured oxidised LDL observed increased plasma levels following six-weeks on a high AGE diet [15]. Standard diets high in AGEs resulted in increased levels of VCAM-1 when compared to a reduced AGE intervention after two-weeks and six-weeks of consumption [24].

#### 3.4.3. Circulating and Excreted CML

Like healthy populations, circulating CML was increased due to a high AGE diet in the majority of studies [15,22,24] in patients with diabetes (Table 4). Calculated effect sizes showed medium to large negative effects on, or increases in circulating AGES due to the high AGE diet and medium to large positive effects, or decreases due to the low AGE diet [19,22]. Urinary CML was measured in only one two-week crossover trial and was reported to be higher following consumption of the high AGE diet suggesting increased excretion [24].

### 3.5. Studies in Patients with CKD

#### 3.5.1. Biomarkers of Inflammation and Oxidative Stress

Consistent with studies in healthy individuals and patients with diabetes, studies in patients with CKD measured TNFα and reported an increase due to a high AGE diet [19,23,25] (Table 2). Effect size could be calculated for only one study [19,23] where the effect of the high AGE diet on TNFα was negligible; while the low AGE diet was found to have a large positive effect [19,23]. Levels of CRP were assessed in one study [19,23] and reported to significantly decrease following the low AGE diet though statistical differences between groups were not described. One study reported a greater decrease in circulating 8-isoprostanes after 4-weeks on a low AGE diet compared with a high AGE [25].

#### 3.5.2. Biomarkers of Chronic Disease Risk

Markers of T2DM risk were not widely assessed in patients with CKD (Table 3). Only one study measured fasting blood glucose and reported no change after four-weeks of dietary intervention [25]. It is not clear whether a high AGE diet increases CVD risk in CKD patients. One study observed an increase in levels of VCAM-1 due to the high AGE diet [19,23] while the other reported no effect [25]. Calculated effect sizes due to high AGE diet were negligible while the low AGE diet had a small positive effect, meaning a reduction in VCAM-1 levels [19,23]. In addition, the effect of the dietary interventions on biomarkers of kidney disease was not clear. Urine albumin and plasma Cystatin C were not reported in either study, while serum creatinine was reported in one study with no differences observed [25].

#### 3.5.3. Circulating and Excreted CML

Again, similar to the studies in other populations, circulating CML was higher in both studies in patients with kidney disease following consumption of a high AGE diet compared to a low AGE intake [19,23,25] (Table 4).

### 3.6. Quality Assessment

The results of the risk of bias assessment and quality assessment are shown in Table 5. In all studies it was not possible to blind the participants to their allocation group as cooking methods were used to produce a difference in AGE content in the diet. As knowledge of treatment group would be unlikely to introduce bias into the outcomes measured, the studies were evaluated based only on the blinding of outcome assessors. One study received a low risk of bias score as defined by a low risk of bias in all key domains [20]. Sources of bias included: not reporting dietary intake [15,21,22,25]; not reporting smoking status [19,22,23,25]; not reporting baseline characteristics or assessing differences between groups at baseline [19,22,23,24,25]; known differences in groups at baseline [17] possible confounding due to weight loss during study [18]; and supplementing both diet groups with beverages high in either fructose or glucose [18].

The quality of the methods employed while conducting the dietary trial was assessed with the quality assessment tool as previously described. Three studies [19,23,25] were found to be of poor quality, including both trials in patients with CKD, due to potential confounding from smoking status not being reported or compliance to dietary intervention not being adequately assessed. The remainder were given a neutral score with not reporting method of randomisation a reoccurring flaw. A summary of overall findings, risk of bias, and quality score is provided in Table 6.

## 4. Discussion

### 4.1. The Effect of a High AGE Diet

This is the first systematic review of RCTs to examine the effect of a high AGE diet on biomarkers of inflammation, oxidative stress and chronic disease risk factors in humans. Unlike previous reviews, this study also considers the effect of a high AGE diet on risk factors for CKD; a highly important consideration given that dietary AGEs may be potentially toxic to the kidneys in individuals susceptible to CKD. The studies presented here used cooking methods to generate differences in the AGE content of interventions, at levels comparable to those found in the Western diet, and so can be considered physiologically relevant. Overall the evidence indicates that consumption of a high AGE diet increases pro-inflammatory biomarkers, specifically TNFα, and circulating levels of AGEs in healthy and overweight individuals and patients with T1DM, T2DM or CKD. The studies reviewed here also suggest that consumption of a high AGE diet may increase biomarkers of oxidative stress in healthy adults, increase cardiovascular risk factors in patients with diabetes, and promote renal dysfunction in overweight males. However, as various methodological issues were identified with several of the studies and high heterogeneity observed between the studies; the results presented here should be interpreted with caution.

There was a lack of evidence surrounding the effect of a high AGE diet on biomarkers of CKD. This is surprising considering the number of studies in patients with CKD [19,23,25] or with diabetes [15,17,22,24] which is a major cause of end-stage renal disease. Overweight and obese, but otherwise healthy individuals are at an increased risk of developing CKD [27]. Therefore, the fact that short-term exposure to a high AGE diet resulted in albuminuria is highly significant. Further studies investigating the effect of a high AGE diet on renal function in healthy populations and individuals with established chronic disease are warranted.

Previous reviews suggest that low AGE intake may be beneficial in reducing biomarkers of inflammation [2,3]. In the current review, the consumption of a high AGE diet for periods greater than two-weeks appeared to increase circulating TNFα levels. This is consistent with studies in cells and in animals which have shown that AGE interaction with the receptor for advanced glycation end-products (RAGE) leads to prolonged activation of nuclear factor kappa B (NFκB) which results in the transcription of pro-inflammatory cytokines, including TNFα [28,29]. Despite an increase in TNFα levels with AGE intake, the same trend was not observed in circulating IL-6 levels. However, IL-6 is known to function as a pro-inflammatory or an anti-inflammatory cytokine depending on the signalling cascade which is activated [30], making these result difficult to interpret. Similarly, the majority of studies presented here also observed no difference in CRP levels. Whilst MCP-1 was measured in in only one short-term study and was increased in response to consumption of a high AGE diet [16]. It is possible that restricting AGE intake could be beneficial in reducing levels of some inflammatory markers, such as TNFα, which could help to prevent or slow the progression of chronic conditions, such as insulin resistance or atherosclerosis [31] however further high quality evidence is required. 

Patients with T2DM are thought to have increased levels of oxidative stress, in particular, of circulating 8-isoprostanes, a marker of lipid peroxidation [32]. Hyperglycaemia induces the generation of reactive oxygen species (ROS) [33,34], promoting the production of 8-isoprostanes through the peroxidation of arachidonic acid [32]. The effect of a high AGE diet on oxidative stress in individuals with diabetes was not well characterised in the included studies. This report suggests that restricting dietary AGE intake in patients with diabetes may be beneficial in reducing levels of oxidative stress. Analysis of the results for the studies involving healthy cohorts [21,22,25] demonstrated that the restricted AGE diet reduced 8-isoprostanes levels, which is in agreement with the findings of an earlier systematic review [2]. Similar to individuals with diabetes, patients with CKD have also been observed to have higher levels of 8-isoprostanes [32] possibly due to impaired renal clearance. Low quality evidence was found which suggests that an AGE restricted diet may lower plasma 8-isoprostanes in patients with CKD. However, only a small number of participants (*n* = 9) were included and therefore, the generalisability of these results are limited. In addition, the authors did not specify whether these patients suffered from comorbidities such as diabetes, which may have confounded results. Again, further higher-quality studies with larger samples sizes are required before a conclusion can be made about the association between a high AGE diet and oxidative stress in chronic conditions. 

All of the studies that reported significant increases in circulating AGEs (measured as CML) in response to a high AGE diet were performed within the same research group [19,21,22,23,24,25]. Before it can be confirmed whether dietary AGEs contribute to elevated plasma levels of AGEs further high quality randomized trials need to be performed by independent research groups. It should be highlighted that there may have been overlap in participants between two 6-week parallel arm intervention studies from the same group of investigators [15,24]. As there were differences in the baseline characteristics reported between articles, the participant groups were treated separately. This may have led to the exaggeration of some of the results obtained in this review. 

Six studies across all populations observed parallel changes in serum AGEs and inflammatory and/or oxidative stress markers [19,21,22,23,24,25], which suggests that AGEs from heat-treated and processed foods are absorbed and enter the circulation where they can drive systemic inflammation. However, two studies reviewed here reported increased levels of pro-inflammatory markers despite not seeing an increase in serum AGEs [16,17]. This gives rise to the idea that a high AGE diet may increase inflammation through mechanisms other than direct absorption into the circulation, such as via effects on gut homeostasis. Few studies in humans have looked at the metabolic fate of dietary AGEs and no studies included in this review measured the AGE content of faecal samples. Recent studies in adolescents suggest that AGEs and other Maillard reaction products may disrupt the composition of the gut microbiota [35]. As changes in microbiota composition are associated with increases in systemic inflammation [36,37] it is possible that the dietary AGEs which escape absorption may also trigger inflammation via this mechanism. Future research in this area should aim to include detailed measurement of the fate of AGEs *in vivo* (*i.e.*, urinary and faecal AGE concentrations or changes to microbiota composition) in order to delineate the physiological mechanisms by which dietary AGEs might elicit their effects. 

Other than inflammatory factors, the studies performed to date suggest that a high AGE diet does not increase risk factors for T2DM in healthy or overweight individuals, patients with diabetes or patients with CKD. Dietary AGEs do not influence fasting blood glucose levels or HbA1_c_ [15,16,17,20,21,22,24,25], however animal studies indicate that dietary AGEs may target pancreatic islets impairing the function of insulin secreting beta cells [38,39,40]. An earlier systematic review [2] reported that there was low-quality evidence [22,41] to indicate that a low AGE diet is beneficial for improving insulin sensitivity in patients with diabetes, yet recent research included in this review does not support this idea [17]. The only short term study [18] that reported reduced insulin sensitivity (HOMA-IR) in healthy women had a high risk of bias and possible confounding due to differences in dietary fat intake between the interventions and weight loss between groups; therefore the impact on insulin sensitivity cannot be conclusively attributed to dietary AGE intake in this study. Over longer periods of AGE consumption [21,22], it appears that it is unlikely that dietary AGEs influence insulin sensitivity, especially in individuals free of chronic disease.

There is evidence to suggest high AGE diets promote risk factors for CVD in patients with diabetes [15,24]. VCAM-1 is an early marker of unstable atherosclerotic plaques [42] and OxLDL is significantly associated with cardiovascular events and stroke in humans [43]. VCAM-1 was improved in response to a low AGE diet [24], while OxLDL was increased following consumption of a high AGE diet in patients with diabetes [15]. This is an important finding as patients with diabetes are at an increased risk of CVD. It appears that reduction of dietary AGEs through simple modification of cooking methods, even in the absence of caloric restriction, may actively reduce CVD risk in these patients. However all studies that measured CVD risk outcomes in this review were from the same research team and before any recommendations can be made these results need to be further verified.

### 4.2. Limitations of Included Studies

The majority of studies included in this review scored low in the quality analysis due to gaps in reporting and methodological flaws, thus limiting the strength of the findings from this review. Only one study reported a power calculation [18] although still failed to achieve the required number of participants to see a significant change in the primary outcome of interest (HOMA-IR) due to drop-outs. As this study had the largest sample size of any study reviewed here, it is therefore highly likely that most studies were underpowered. Executing controlled dietary studies with free-living participants is incredibly difficult due to economic constraints, and burden on the participants, and these factors likely contributed to the small sample sizes in the included studies.

The diversity of study populations further complicated the interpretation of this review. Medication use of participants with diabetes included statins [15,24], metformin, sulfonylureas [17] or insulin [24], while some were underwent dietary therapy alone [15,17,24]. Whether the use of medications affected the outcomes of interest is unclear, but is a potential confounding factor. As with all studies in populations with insulin resistance or diabetes, the variation in medications use treatments indicate that participants are likely to have different levels of glucose control and less likely to be comparable in terms of their physiological responses. The heterogeneity within and between these studies severely limits any conclusions surrounding the effect of a high AGE diet in patients with diabetes.

Though there were several studies which measured the effect of a high AGE diet on markers of inflammation and oxidative stress, and serum AGEs, due to differences in the way the results were reported and also missing n values for intervention groups in one study [25], it was not possible to perform a meta-analysis on the studies in this review. 

Some studies reported use of vitamin supplements [15,24] while others did not [17,19,21,22,23,25] or asked participants to refrain from taking any supplements during the intervention period [16,18,20]. Vitamin B6 is a known AGE inhibitor [29] therefore failure to control for supplement intake could confound results. Several studies failed to report smoking status of the participants and did not list smoking as exclusion criteria [19,22,23,25]. This would represent a major confounder as tobacco smoke is another source of exogenous AGEs [44].

Dietary intake was poorly reported in several articles [15,21,22,25]. Some studies that relied on participants to prepare their own food reported significant differences in protein or fat intake between intervention groups [18,19,23]. Therefore in the studies that are missing dietary intake information and that did not provide food to participants [21,22,25] it is likely that there may have been differences other than the AGE content between interventions. Designing isocaloric diets that are matched for macronutrient and micronutrient content but contain varying AGE levels generated through cooking methods is challenging. However with thorough planning and controlled portion sizes confounding factors can be minimised [45]. Providing fully prepared meals to the participants adds significant strength to research into dietary AGEs however less than half of the studies reported here used this approach [15,16,17,20,24].

The majority of studies relied on a published AGE database [26] to determine the AGE content of the diet. However this database was generated using an enzyme linked immunosorbent assay (ELISA) that had not been validated against LC-MS and therefore may overestimate or underestimate the true AGE content of a food substance [46]. There is a database [10] of the AGE content of foods measured by LC-MS now available, although only a limited number of foodstuffs have been analyzed. Future research in this area should use databases which have AGEs measured by validated techniques.

Finally, the included studies had relatively short follow up duration (two to 16-weeks). The adverse consequences of a high AGE diet may arise over a period of years rather than weeks or months as AGEs accumulate, glycate other proteins, or are incorporated into tissues. Therefore, studies of longer duration are required.

### 4.3. Limitations of this Review

The risk of bias assessment tool used in this review used may have resulted in higher risk of bias or poorer quality ranking being assigned to studies that were more transparent with reporting. Also, limitation of the included studies to isocaloric diets resulted in the exclusion of one relevant and well reported trial [41]. Finally, the outcomes assessed here are biomarkers and do not necessarily predict end points or disease outcomes. Longer-term trials that include disease outcome are required before the effect of a high AGE diet on incidence of chronic disease can be confirmed. Also, a major concern highlighted in this review is that eight [15,19,21,22,23,24,25] of the included studies were performed by the same research group and therefore the external validity of the results of these studies is questionable. This then limits the generalisabilty of this review.

### 4.4. Comparison with Other Reviews

The conclusions drawn about the lack of high quality long-term trials in this field agree with previously published systematic reviews [2,3] despite limiting included studies to randomised controlled trials. The three most recent interventions [18,20,21] published after the earlier reviews were of variable quality. One of these recent studies [20] which was found to have a low risk of bias, addressed many of the issues highlighted by Kellow *et al.* [2]. Another, however, had major methodological flaws and confounding factors which resulted in a high risk of bias rating [18]. The overall findings that a high AGE diet may increase circulating TNFα, oxidative stress and circulating AGEs corroborate those reported in earlier systematic reviews and support the need for future high quality research in this area.

## 5. Conclusions

The findings of this review suggest that consumption of a high AGE diet increases circulating levels of TNFα and AGEs in healthy individuals and in individuals with chronic disease. Furthermore, there is evidence to suggest that dietary AGEs promote oxidative stress in healthy adults, and increase CVD markers in patients with diabetes. As such, dietary AGEs may play a role in the promotion of chronic conditions such as T2DM, CVD and CKD through increasing oxidative stress and inflammation.

The limitations of the current evidence, highlighted in this review, indicate that further high quality randomised controlled trials are required to fully delineate the adverse consequences of a high AGE diet in both healthy people and in patients with diabetes and CKD before any dietary recommendations can be made. Future studies into the effect of a high AGE diet, or benefit of a low AGE diet need to: (i) control for confounding factors (such as fat content or heat sensitive vitamins) between study diets; (ii) use validated methods to assess the AGE content of foods in the diet; (iii) provide participants with meals to increase compliance; (iv) include justifications of sample size; (v) include biomarkers of kidney function as primary outcomes; (vi) include methods to assess the metabolism of dietary AGEs in order to better elucidate the mechanisms by which a high AGE diet may have an adverse effect. Given that the standard Western diet contains a high amount of AGEs, further research into the consequences of habitual high AGE diets is important. The potential benefits of restricted AGE intake are promising and could offer a simple dietary therapy in the prevention and treatment of chronic conditions. 

## Figures and Tables

**Table 1:** Characteristics of included dietary interventions.

Study	Intervention	Comparator	Length (Weeks)	AGE Content of Diet	Assessment of AGEs	Participants Intervention Comparator	
Harcourt *et al.* 2011 ^a^ [16]	High AGE diet. Food provided. Cooking methods used to generate difference in AGEs. P:F:C = 16:30:54	Low AGE diet. Food provided. Cooking methods used to generate difference in AGEs. P:F:C = 16:30:54	2	H-AGE = 14,090, L-AGE = 3302 kU AGE/day	Based on reference database not validated [26]	Healthy overweight: *n* = 11 (M = 100%); Age = 30 ± 9 years; BMI = 31.8 ± 4.8 kg/m^2^.	
Mark *et al.* 2014 ^b^ [18]	High AGE diet. Ingredients provided and participants instructed on how to prepare meals. Cooking methods used to generate difference in AGEs. P:F:C = 18.8 ± 0.4:37.3 ± 0.8:42.7 ± 0.9	Low AGE diet. Food provided. Cooking methods used to generate difference in AGEs. P:F:C = 21.6 ± 0.4:30.6 ± 0.7:46.9 ± 0.8	4	H-AGE = 24.6; L-AGE = 10.7 mg/day CML (H-AGE had 43% more AGEs)	LC-MS	Healthy overweight: *n* = 37 (M = 0); Age = 41.4 ± 1.4 years; BMI = 32.3 ± 0.6 kg/m^2^	Healthy overweight: *n* = 36 (M = 0); Age = 37.9 ± 1.4 yrs; BMI = 33.2 ± 0.8 kg/m^2^
Semba *et al.* 2014 ^b^ [20]	High AGE diet. Food provided. Cooking techniques and time used to produce AGEs. P:F:C = 17:29:55 *	Low AGE diet. Food provided. Cooking technique and time varied to reduce AGEs.	6	H-AGE = 4 times AGE content of L-AGE	Based on reference database not validated [26]	Healthy: *n* = 12 (M = 41.7%); Age = 60.6 ± 4.3 yrs; BMI = 26.1 ± 3.4 kg/m^2^	Healthy: *n* = 12 (M = 41.7%); Age = 57.9 ± 6.0 yrs; BMI = 26.4 ± 4.0 kg/m^2^
Uribarri *et al.* 2011 ^b^ [22]	Standard diet high in AGES. Participants prepared own food. Cooking techniques and time used to produce AGEs.	Low AGE diet. Participants prepared own foods under instruction. Cooking technique and time varied to reduce AGEs.	16	L-AGE = 40%–50% reduction in AGEs compared to H-AGE	Based on reference database not validated	Healthy: *n* = 9 (M = 22.2%); Age = 67 ± 1 years; BMI = 27.3 ± 1.4 kg/m^2^	Healthy: *n* = 9 (M = 22.2%); Age = 67 ± 1 years; BMI = 27.3 ± 1.4 kg/m^2^
Uribarri *et al.* 2014 ^b^ [21]	Standard diet high in AGES. Participants prepared own food. Cooking techniques and time used to produce AGEs.	Low AGE diet. Participants prepared own foods under instruction. Cooking technique and time varied to reduce AGEs.	16	H-AGE ≥ 15; L-AGE < 10 kU AGE/day	Based on reference database not validated	Healthy: *n* = 8 (M = 25%); Age = 63.5 ± 5 years; BMI = 29 ± 2 kg/m^2^	Healthy: *n* = 10 (M = 30%); Age = 65 ± 2 years; BMI = 26 ± 3 kg/m^2^
Vlassara *et al.* 2009 ^b^ [25]	Standard diet high in AGES. Participants prepared own food. Cooking techniques and time used to produce AGEs.	Low AGE diet. Participants prepared own foods under instruction. Cooking technique and time varied to reduce AGEs.	16	H-AGE > 20,000; L-AGE < 10,000 kU CML/day	Based on reference database not validated [26]	30 Healthy participants randomised to either H-AGE or L-AGE diet. BMI = 28 ± 2 kg/m^2^ Further population characteristics not described.	
Cai *et al.* 2004 ^b^ [15]	High AGE diet. Food provided. Cooking methods used to generate difference in AGEs. P:F:C = 20:30:50	Low AGE diet. Food provided. Cooking technique and time varied to reduce AGEs. P:F:C = 20:30:50	6	H-AGE = 16300 ± 3700; L-AGE = 3670 ± 1200 kU CML/day	Competitive ELISA for protein foods, direct ELISA for lipid foods. Not validated	T1DM and T2DM: *n* = 11 (M = 54%); Age = 61 ± 7 years; BMI = 28.2 ± 3.4 kg/m^2^; T1DM:T2DM = 2:9	T1DM and T2DM *n* = 13 (M = 38%); Age = 62 ± 5 years; BMI = 28.7 ± 5.1 kg/m^2^; T1DM:T2DM = 4:9
Luevano-Contreras *et al.* 2013 ^b^ [17]	Standard diet high in AGEs. Participants prepared own food. Cooking technique and time used to produce AGEs. P:F:C = 20:30:50	Low AGE diet. Participants prepared own foods under instruction. Cooking technique and time varied to reduce AGEs. P:F:C = 20:30:50	6	H-AGE = 9910 ± 4169; L-AGE = 8956 ± 3587 kU CML/day (from baseline)	Based on reference database not validated [26]	T2DM: *n* = 13 (M = 15.4%); Age = 48.5 ± 6.2 years; BMI = 28.8 ± 4. kg/m^2^	T2DM: *n* = 13 (M = 7.7%); Age = 46.0 ± 5 years; BMI = 29.8 ± 4.0 kg/m^2^
Uribarri *et al.* 2011 ^b^ [22]	Standard diet high in AGES. Cooking technique and time used to produce AGEs. Participants prepared own food.	Low AGE diet. Participants prepared own foods under instruction. Cooking technique and time varied to reduce AGEs.	16	L-AGE = 40%–50% reduction in AGEs compared to H-AGE	Based on reference database not validated	T2DM: *n* = 6 (M = 22.2%); Age = 61 ± 4 years; BMI = 32.3 ± 1.6 kg/m^2^	T2DM: *n* = 12 (M = 22.2%); Age = 61 ± 4 years; BMI = 32.3 ± 1.6 kg/m^2^
Vlassara *et al.* 2002 ^b^ [24]	High AGE diet. Food provided. Cooking techniques and time used to produce AGEs. P:F:C = 20:30:50	Low AGE diet. Food provided. Cooking technique and time varied to reduce AGEs. P:F:C = 20:30:50	6	H-AGE = 16,300 ± 3700; L-AGE = 3670 ± 1200 kU CML/day	Competitive ELISA for protein foods, direct ELISAs for lipid foods. Not validated	T1DM and T2DM: *n* = 6 Age = 62 years; BMI = 29.5 ± 3 kg/m^2^ T1DM:T2DM = 4:8	T1DM and T2DM: *n* = 7 Age = 62 years; BMI = 29.5 ± 3 kg/m^2^
Vlassara *et al.* 2002 ^a^ [24]	High AGE diet. Food provided. Cooking methods used to generate difference in AGEs. P:F:C = 20:30:50	Low AGE diet. Cooking technique and time varied to reduce AGEs. P:F:C = 20:30:50	2	H-AGE = 16,300 ± 3700 L-AGE = 3670 ± 1200 kU CML/day	Competitive ELISA for protein foods, direct ELISAs for lipid foods. Not validated	T1DM and T2DM: *n* = 11 Age = 52 ± 5 years; BMI = 28 ± 3.5 kg/m^2^ T1DM:T2DM = 2:9	
Uribarri 2003; Peppa 2004 ^b^ [19,23]	Standard diet high in AGEs. Participants prepared own food. Cooking techniques and time used to produce AGEs.	Low AGE diet. Cooking technique and time varied to reduce AGEs. Participants prepared own foods under instruction	4	H-AGE = 17,000 ± 3700; L-AGE = 5500 ± 900 kU CML/day	Based on reference database not validated [26]	Non diabetic peritoneal dialysis; *n* = 9 (M = 33.33%). Not significantly different from comparator at baseline.	Non diabetic peritoneal dialysis; *n* = 9 (M = 33.33%)
Vlassara *et al.* 2009 ^b^ [25]	Standard diet high in AGES. Participants prepared own food. Cooking techniques and time used to produce AGEs.	Low AGE diet. Meals prepared in the clinical research center and given to participants twice a week. Cooking technique and time varied to reduce AGEs.	4	H-AGE > 20,000; L-AGE < 10,000 kU CML/day	Based on reference database not validated [26]	9 CKD (stage 3) patients randomised to either H-AGE or L-AGE diet. BMI = 23 ± 1.6 kg/m^2^ (intervention); 28 ± 1.9 kg/m^2^ (comparator). Further population characteristics not described	

AGE = Advanced glycation end-product; BMI = body mass index; CML = carboxymethyl lysine; CKD = Chronic kidney disease; CRP = C-reactive protein; ELISA = Enzyme linked immunosorbent assay; FBG = fasting blood glucose; H-AGE = high AGE diet; HbA1_c_ = glycated haemoglobin; HOMA IR = homeostatic model assessment insulin resistance; IL-6 = Interleukin 6; kU = kilo units; L-AGE = low AGE diet; LC-MS = Liquid chromatography- mass spectrometry; M = male; MCP-1 = monocyte chemoattractant protein-1; ND = Not described; P:F:C = percentage of total energy from protein, fat and carbohydrate of diet; PBMC = peripheral blood mononuclear cells; T1DM = type 1 diabetes mellitus; T2DM = type 2 diabetes mellitus; TNFα = tumour necrosis factor α; VCAM-1 = vascular cell adhesion molecule 1. Data reported as mean ± standard deviation; ^a^ randomised cross over trial; ^b^ randomised parallel arm trial; * Calculated from protein, fat and carbohydrate intake in grams/day.

**Table 2:** Circulating biomarkers of inflammation and oxidative stress.

Study	Group	*n*	TNFα	ES	IL-6	ES	CRP	ES	MCP-1	ES	8-Isoprostane	ES
*Healthy*												
**Harcourt *et al.* 2011 [16]**	L-AGE H-AGE	11CO			NVG		NVG		**H > L (plasma)**		**H > L (urine)**	
**Semba *et al.* 2014 [20]**	L-AGE	12			B: 1.48 (1.84) A: 1.53 (1.35) pg/mL	−0.03	B: 2.11 (1.45) A: 2.62 (2.25) mg/L	−0.27				
	H-AGE	12			B: 2.25 (1.84) A: 2.09 (1.35) pg/mL	+0.10	B: 1.57 (1.45) A: 1.38 (2.25) mg/L	+0.10				
**Uribarri *et al.* 2011 [22]**	L-AGE	9	**B: 10 (3.9)** **A: 8.4 (2.1)** **ng/mg (PBMC)**	+0.51						****	**B: 135 (30)** **A: 90 (27)** **pg/mL (plasma)**	+1.58
	H-AGE	9	**B: 8.6 (1.8)** **A: 11.8 (3)** **ng/mg (PBMC)**	−1.29							**B: 125 (54)** **A: 165 (69)** **pg/mL (plasma)**	−0.65
**Uribarri *et al.* 2014** [21]	L-AGE	10	MD: −2.1 (1.6) * ng/mg (PBMC)				MD: 1.3 (1.4) * mg/L			****	**B: 170 (65)** **A: 85 (17)** **MD:** −48 (11) * pg/mL (serum)	+1.79
	H-AGE	8	MD: +3.2 (0.8) * ng/mg (PBMC)				MD: 0.4 (0.4) * mg/L				**MD: +48 (20)** pg/mL (serum) *	
**Vlassara *et al.* 2009 [25]**	L-AGE	30	B: 12 (1) * A: 8 (0.5) * **ng/mg (PBMC)**								B: 240 (67) * A: 100 (13) * ng/mL (plasma)	-
	H-AGE		B: 9 (1) * A: 12 (1) * ng/mg (PBMC)								B: 122 (4) * A: 173 (38) * ng/mL (plasma)	
***Diabetes***												
**Luevano-Contreras *et al.* 2013 [17]**	L-AGE	13	MD: −18.36 (17.1) * pg/mL (serum)				MD: −1.69 (5.4) * mg/L (serum)					
	H-AGE	13	MD: +12.5 (14.7) * pg/mL (serum)				MD: −1.21 (5.5) * mg/L (serum)					
**Uribarri *et al.* 2011 [22]**	L-AGE	12	**B: 18 (3.5)** **A: 14.4 (6.9)** **ng/mg (PBMC)**	+0.66							B: 233 (58.9) A: 141 (62.4) pg/mL (plasma)	+1.52
	H-AGE	6	**B: 20 (4.9)** **A: 26 (4.9)** **ng/mg (PBMC)**	−1.22							B: 236 (61.2) A: 313 (188.6) pg/mL (plasma)	−0.55
**Vlassara *et al.* 2002 6-weeks [24]**	L-AGE	7	MD:−20% * ng/mL (PBMC)				MD: −20% * mg/dL (serum)					
	H-AGE	6	MD: +86.3% * ng/mL (PBMC)				MD: +35% * mg/dL (serum)					
**Vlassara *et al.* 2002 2-weeks [24]**	L-AGE	11CO					MD: 4.1 (4.8) * mg/dL (serum)					
	H-AGE						MD: 6 (8.6) * mg/dL (serum)					
***Kidney Disease***												
**Uribarri *et al.* 2003; ** **Peppa 2004 *et al.* [19,23]**	L-AGE	9	**B: 44 (18)** **A: 31 (10.8)** **pg/mg (PBMC)**	+0.88			**Graph only**					
	H-AGE	9	**B: 43 (21)** **A: 44 (21)** **pg/mg (PBMC)**	−0.05			Graph only					
**Vlassara *et al.* 2009 [25]**	L-AGE	9	B: 22 (4) * A: 16 (3) * ng/mg (PBMC)								B: 328 (51) * A: 154 (25) * ng/mL (plasma)	
	H-AGE		B: 15 (4) * A: 16 (3) * ng/mg (PBMC)								B: 211 (7) * A: 167 (21) * ng/mL (plasma)	

*Abbreviations:* A = after intervention; AGE = Advanced glycation end-product; B = baseline; CO = Crossover trial; CRP = C reactive protein; ES = Effect Size, H-AGE = high AGE diet; IL-6 = Interleukin-6; L-AGE = low AGE diet; MCP-1 = monocyte chemoattractant protein-1; MD = mean difference from baseline; NVG = no value give; PBMC = peripheral blood mononuclear cells; TNFα = tumour necrosis factor α. Data reported as mean (standard deviation) except where * indicates mean (standard error of the mean). Bold indicates significant differences between groups or from baseline (*p* < 0.05). Bold indicates significant differences between groups or from baseline (*p* < 0.05).

**Table 3:** Biomarkers of chronic disease risk.

Study	Group	*n*	T2DM						CVD				CKD			
			HOMA IR	ES	FBG	ES	HbA1_c_ %	ES	oxLDL	ES	VCAM-1	ES	Alb	ES	Cr	ES
***Healthy***																
**Harcourt *et al.* 2011 [16]**	L-AGE	11CO			A: 5.1 (0.3) mmol/L								**H > L**		A: 72.3 (18.3) μmol/L (serum)	
	H-AGE															
**Mark *et al.*** **2014 [18]**	L-AGE	36	**B: 2.65 (1.8)** **A: 2.43 (1.8)**	+0.12	B: 5.4 (0.6) A: 5.5 (0.6) mmol/L	−0.17										
	H-AGE	37	**B: 2.14 (1.8)** **A: 2.40 (1.2)**	−0.17	B: 5.5 (0.61) A: 5.5 (0.61) mmol/L	0										
**Semba *et al.*** **2014 [20]**	L-AGE	12			B: 97 (10.4) A: 93 (10.4) mg/dL	+0.38					B: 1.26 (0.8) A: 1.01 (0.9) ug/mL	+0.29				
	H-AGE	12			B: 93 (10.4) A: 96 (10.4) mg/dL	−0.29					B: 1.39 (0.8) A: 1.34 (0.9) ug/mL	−0.06				
**Uribarri *et al.*** **2011 [22]**	L-AGE	9	B: 2.2 (0.9) A: 2.5 (1.8)	−0.33	B: 86 (9) A: 88 (18) mg/dL	−0.14										
	H-AGE	9	B: 1.3 (0.9) A: 1.5 (1.8)	−0.22	B: 83 (12) A: 80 (12) mg/dL	+0.25										
**Uribarri *et al.*** **2014 [21]**	L-AGE	10	MD: 0.04 (0.44) *								MD: −270 (92) * pg/mL **(serum)**					
	H-AGE	8	MD: 0.14 (0.15) *								MD:+182 (46) * pg/mL **(serum)**					
**Vlassara *et al.*** **2009 [25]**	L-AGE	30			B: 81 (3) * A: 78 (3) * mg/dL						B: 1001 (115) * A: 576 (49) * ng/mL (plasma)				B: 99 (12) * A: 118 (15) * mL/min (Cr clearance)	
	H-AGE				B: 84 (3) * A: 79 (3) * mg/dL						B: 717 (68) * A: 771 (58) * ng/mL (plasma)				B: 115 (10) * A: 100 (8) * mL/min (Cr clearance)	
***Diabetes***																
**Cai *et al.*** **2004 [15]**	L-AGE	13			B: 122 (90.1) A: 118 (68.5) mg/dL	+0.05	B: 7.2 (3.6) A: 7.0 (2.88)	+0.06	**A:1.5 (0.5)** **nmol/mg**							
	H-AGE	11			B: 116 (146.6) A:128 (71.6) mg/dL	−0.10	B: 7.3 (2.0) A: 7.4 (4.3)	−0.03	**A:5.7 (2.3)** **nmol/mg**							
**Luevano-Contreras** ***et al.* 2013** [17]	L-AGE	13	MD: −2.29 (3.7) *		MD: −18 (56.7) * mg/dL		MD: 0.19 (1.3) *									
	H-AGE	13	MD: −2.5 (6.1)*		MD: 4.55 (35.6) * mg/dL		MD: −0.11 (1.9) *									
**Uribarri *et al.*** **2011 [22]**	L-AGE	12	**B: 5.3 (1.4)** **A: 3.4 (2.1)**	+1.06	B: 114 (24.2) A: 111 (31.2) mg/dL	+0.11	B: 6.4 (0.7) A: 6.6 (1.4)	−0.18								
	H-AGE	6	**B: 4.5 (2.9)** **A: 6.2 (1.2)**	−0.77	B: 131 (90) A: 129 (63.7) mg/dL	+0.03	B: 6.7 (1.2) A: 6.5 (1.0)	+0.18								
**Vlassara *et al.*** **2002 6-weeks** [24]	L-AGE	7			B: 7.0 (2.7) A: 5.6 (1.3) mmol/L	+0.68					MD: −20% * ng/mL (serum)					
	H-AGE	6			B: 6.5 (2.9) A: 8.1 (2.7) mmol/L	−0.57										
**Vlassara *et al.*** **2002 2-weeks [24]**	L-AGE	11CO			A: 7.5 (0.7) * mmol/L						MD:698 (347) * ng/mL **(serum)**					
	H-AGE				A: 8.1 (0.4) * mmol/L						MD:1108 (429) * ng/mL **(serum)**					
***Kidney Disease***																
**Uribarri *et al.* 2003; Peppa 2004 *et al.* [19,23]**	L-AGE	9									**B: 3448 (483)** **A: 3244 (708)** **ng/mL (serum)**	+0.34				
	H-AGE	9									**B: 3699 (306)** **A: 3735 (291)** **ng/mL** **(serum)**	−0.12				
**Vlassara *et al.* 2009 [25]**	L-AGE	9			B: 89 (3) * A: 90 (3) * mg/dL						B: 1033 (168) * A: 733 (52) * (ng/mL) (plasma)				B: 39.5 (11) * A: 36 (10) * mL/min (Cr clearance)	
	H-AGE				B: 93 (2) * A: 80 (4) * mg/dL						B: 1086 (210) * A: 956 (133) * (ng/mL) (plasma)				B: 46.5 (15) * A: 43 (11) * mL/min (Cr clearance)	

*Abbreviations:* A = after intervention; AGE = Advanced glycation end-product; Alb = Albuminuria; B = baseline; CO = Crossover trial; Cr = creatinine; ES = Effect Size; FBG = fasting blood glucose; H-AGE = High AGE diet; HbA1_c_ = glycated haemoglobin; HOMA IR = homeostatic model assessment insulin resistance; L-AGE = Low AGE diet; MD = mean difference from baseline; NS = not significant; oxLDL = oxidised low density lipoprotein; VCAM-1 = vascular cell adhesion molecule 1. Data are reported mean (standard deviation) except where * indicates mean (standard error of the mean). Bold indicates significant differences between groups (*p* < 0.05). Data reported with significant figures as described in publication. Bold indicates significant differences between groups or from baseline (*p* < 0.05).

**Table 4:** Circulating levels and urinary excretion of the advanced glycation end-product Carboxymethyl Lysine (CML).

Study	Group	*n*	Circulating CML	ES	Urinary CML	ES
*Healthy*						
**Harcourt *et al.* 2011 [16]**	L-AGE H-AGE	11CO	**L > H**	****	**H > L**	
**Mark *et al.* 2014 [18]**	L-AGE H-AGE	36 37			**H > L**	
**Semba *et al.* 2014 [20]**	L-AGE	12	B: 763 (83) A: 678 (100) ng/mL (serum)	+0.92	B: 1.37 (5.10) A: 0.77 (6.96) μg/mg creatinine	+0.10
	H-AGE	12	B: 751 (83) A: 711 (100) ng/mL (serum)	+0.44	B: 1.03 (5.10) A: 1.21 (6.96) μg/mg creatinine	−-0.03
**Uribarri *et al.* 2011 [22]**	L-AGE	9	**B: 12.4 (1.5)** **A: 9.3 (3.0) U/mL (serum)**	+1.31		
	H-AGE	9	**B: 11.7 (3.9)** **A: 14.0 (3.0) U/mL (serum)**	-0.66		
**Uribarri *et al.* 2014 [21]**	L-AGE	10	**B: 13.7 (3.2) A: 9.2 (2.5)** MD: −3.71 (1.03) * U/mL (serum)	+1.57		
	H-AGE	8	MD: +1.87 (1.05) * U/mL serum)	-		
**Vlassara *et al.* 2009 [25]**	L-AGE	30	B: 14 (1) * A: 9 (1) * U/mL (serum)	-		
	H-AGE		B: 11 (1) * A: 13 (1) * U/mL (serum)			
*Diabetes*						
**Cai *et al.* 2004 [15]**	L-AGE	13	**B: 12.5 (7.9)** **A: 7.9 (4.0) U/mL (serum)**	+0.73		
	H-AGE	11	**B: 13.1 (8.6)** **A: 18.0 (5.6) U/mL (serum)**	−0.68		
**Uribarri *et al.* 2011 [22]**	L-AGE	12	**B: 17.1 (4.5)** **A: 11.6 (3.8) U/mL (serum)**	+1.32		
	H-AGE	6	**B: 17.8 (4.9)** **A: 24.2 (9.8) U/mL (serum)**	−0.83		
**Study**	**Group**	**n**	**Circulating CML**	**ES**	**Urinary CML**	**ES**
**Vlassara *et al.* 2002** **6-weeks [24]**	L-AGE	7	**MD: −40% U/mL (serum)**			
	H-AGE	6	**MD: 28.2 % U/mL (serum)**			
**Vlassara *et al.* 2002** **2-weeks [24]**	L-AGE	11CO	A: 7.7 (2.4) * U/mL (serum) A: 13 (6) * U/mL (serum)		**A: 15.26 (10) × 10^−3^ U/24 h** **A: 30.4 (12) × 10^−3^ U/24 h**	
	H-AGE					
***Kidney Disease***						
**Uribarri *et al.* 2003;** **Peppa 2004 *et al.* [19,23]**	L-AGE	9	**MD: −34% (serum)**			
	H-AGE	9	**MD: 29% (serum)**			
**Vlassara *et al.* 2009 [25]**	L-AGE	9	B: 25 (3) * A: 14.2 (2) * U/mL (serum)			
	H-AGE		B: 19 (4) * A: 17 (3) * U/mL (serum)			

*Abbreviations:* A = after intervention; AGE = Advanced glycation end-product; B = baseline; CML = carboxymethyl lysine; CO = crossover trial; ES = Effect Size; H-AGE = high AGE diet; L-AGE = low AGE diet; MD = mean difference from baseline; N/A = Not assessed; U = units. Data reported as mean (standard deviation) except where * indicates mean (standard error of the mean). − = insufficient data to calculate effect size. Bold indicates significant differences between groups (*p* < 0.05). Data reported with significant figures as described in publications. Bold indicates significant differences between groups or from baseline (*p* < 0.05).

**Table 5:** Assessment of studies using Cochrane risk of bias tool and the American Dietetic Association quality criteria checklist.

	Adequate Sequence Generation	Adequate Allocation Concealment	Blinding- Outcome Assessors	Incomplete Outcome Data Addressed	Free of Selective Outcome Reporting	Free of Other Bias	Overall Risk of Bias	Quality (+, −, or Neutral)
*Healthy*								
Harcourt *et al.* 2011 [16]	Unclear	Unclear	Unclear	+	Unclear	+	Unclear	Neutral
Mark *et al.* 2014 [18]	Unclear	Unclear	-	+	Unclear	–(weight loss occurred and supplemented with high fructose or glucose beverages)	High	Neutral
Semba *et al.* 2014 [20]	+	+	+	+	+	+	Low	Neutral
Uribarri *et al.* 2011 [22]	Unclear	Unclear	Unclear	+	Unclear	Unclear (differences in intervention groups at baseline, smoking and dietary intakes not reported)	Unclear	Neutral
Uribarri *et al.* 2014 [21]	Unclear	Unclear	Unclear	+	+	Unclear (dietary intake not reported)	Unclear	Neutral
Vlassara *et al.* 2009 [25]	Unclear	Unclear	Unclear	+	Unclear	Unclear (differences in intervention groups at baseline, smoking and dietary intakes not reported)	Unclear	-
*Diabetes*								
Cai *et al.* 2004 [15]	Unclear	Unclear	Unclear	+	Unclear	Unclear (dietary intake not reported)	Unclear	Neutral
Luevano- Contreras *et al.* 2013 [17]	+	+	+	+	Unclear	–(difference in TNFα levels between groups at baseline)	High	Neutral
Vlassara *et al.* 2002 2-weeks [24]	Unclear	Unclear	Unclear	+	-	Unclear (baseline characteristics not reported in detail)	High	Neutral
Vlassara *et al.* 2002 6-weeks [24]	Unclear	Unclear	Unclear	Unclear	Unclear	Unclear (baseline characteristics not reported)	Unclear	Neutral
*Kidney Disease*								
Uribarri *et al.* 2003/Peppa *et al.* 2004 [19,23]	Unclear	Unclear	Unclear	+	Unclear	Unclear (intervention group significantly increased caloric intake from baseline, smoking not reported)	Unclear	-
Vlassara *et al.* 2009 [25]	Unclear	Unclear	Unclear	+	Unclear	Unclear (differences in intervention groups at baseline, smoking and dietary intakes not reported)	Unclear	-

*Abbreviations:* TNFα = Tumour necrosis factor alpha. + = Yes/ Free of bias/high quality; − = No/risk of bias/poor quality.

**Table 6:** Summary of findings from included studies.

Study	Length (weeks)	Population	Inflammation	Oxidative Stress	T2DM Risk	CVD Risk	CKD Risk	cAGEs	uAGEs	Risk of Bias	Quality
*Healthy*											
Harcourt *et al.* 2011 [16]	2	Healthy overweight	↑/↔	↑	↔	N/A	↑/↔	↓	↑	Unclear	Neutral
Mark *et al*. 2014 [18]	4	Healthy overweight	N/A	N/A	↑/↔	N/A	N/A	N/A	↑	High	Neutral
Semba *et al.* 2014 [20]	6	Healthy	↔	N/A	↔	↔	N/A	↔	↔	Low	Neutral
Uribarri *et al.* 2011 [22]	16	Healthy	↑	↑	↔	N/A	N/A	↑	N/A	Unclear	Neutral
Uribarri *et al.* 2014 [21]	16	Healthy	↑/↔	↑	↔	↑	N/A	↑	N/A	Unclear	Neutral
Vlassara *et al.* 2009 [25]	16	Healthy	↑	↔	↔	↔	N/A	↑	N/A	Unclear	-
*Diabetes*											
Cai *et al.* 2004 [15]	6	T1DM + T2DM	N/A	N/A	↔	↑	N/A	↑	N/A	Unclear	Neutral
Luevano-Contreras *et al.* 2013 [17]	6	T2DM	↑/↔	N/A	↔	N/A	N/A	↔	N/A	High	Neutral
Uribarri *et al.* 2011 [22]	16	T2DM	↑	↑	↑/↔	N/A	N/A	↑	N/A	Unclear	Neutral
Vlassara *et al.* 2002 [24]	6	T1DM + T2DM	↑	N/A	↑	↑	N/A	↑	↑	High	Neutral
Vlassara *et al.* 2002 [24]	2	T1DM + T2DM	↔	N/A	↑	↑	N/A	↑	N/A	Unclear	Neutral
*Kidney Disease*											
Uribarri *et al.* 2003; Peppa 2004 [19,23]	4	CKD	↑	N/A	N/A	↑	N/A	↑	N/A	Unclear	-
Vlassara *et al.* 2009 [25]	4	CKD	N/A	↑	↔	↔	↔	↑	N/A	Unclear	-

*Abbreviations:* AGE = Advanced glycation end-product; cAGEs = circulating AGEs; CKD = Chronic kidney disease; CVD = cardiovascular disease; N/A = Not assessed; T2DM = type 2 diabetes mellitus; uAGEs = urinary AGEs. ↑ = significantly increased following a high AGE diet or standard diet high in AGEs when compared to a low AGE diet; ↔ = Negligible effect size and/or no differences in outcome between a high AGE and low AGE diet; ↓ = significantly decreased; ↓ significantly decreased following a high AGE diet or standard diet high in AGEs when compared to a low AGE diet.

## References

[1] Beaglehole R., Bonita R., Horton R., Adams C., Alleyne G., Asaria P., Baugh V., Bekedam H., Billo N., Casswell S. (2011). Priority actions for the non-communicable disease crisis. Lancet..

[2] Kellow N.J., Savige G.S. (2013). Dietary advanced glycation end-product restriction for the attenuation of insulin resistance, oxidative stress and endothelial dysfunction: A systematic review. Eur. J. Clin. Nutr..

[3] Van Puyvelde K., Mets T., Njemini R., Beyer I., Bautmans I. (2014). Effect of advanced glycation end product intake on inflammation and aging: A systematic review. Nutr. Rev..

[4] Feng J.X., Hou F.F., Liang M., Wang G.B., Zhang X., Li H.Y., Xie D., Tian J.W., Liu Z.Q. (2007). Restricted intake of dietary advanced glycation end products retards renal progression in the remnant kidney model. Kidney Int..

[5] Šebeková K., Faist V., Hofmann T., Schinzel R., Heidland A. (2003). Effects of a diet rich in advanced glycation end products in the rat remnant kidney model. Am. J. Kidney Dis..

[6] ŠEbekovÁ K., Hofmann T., Boor P., UlicnÁ O.G., Erbersdobler H.F., Baynes J.W., Thorpe S.R., Heidland A., Somoza V. (2005). Renal effects of oral Maillard reaction product load in the form of bread crusts in healthy and subtotally nephrectomized rats. Ann. N. Y. Acad. Sci..

[7] Zheng F., He C., Cai W., Hattori M., Steffes M., Vlassara H. (2002). Prevention of diabetic nephropathy in mice by a diet low in glycoxidation products. Diabetes Metab. Res. Rev..

[8] Somoza V., Lindenmeier M., Hofmann T., Frank O., Erbersdobler H.F., Baynes J.W., Thorpe S.R., Heidland A., Zill H., Bek S. (2005). Dietary bread crust advanced glycation end products bind to the receptor for AGEs in HEK-293 kidney cells but are rapidly excreted after oral administration to healthy and subtotally nephrectomized rats. Ann. N. Y. Acad. Sci..

[9] World Health Organisation (2015). Healthy Diet Fact Sheet.

[10] Dresden University of Technology (2012). AGE Database.

[11] Moher D., Shamseer L., Clarke M., Ghersi D., Liberati A., Petticrew M., Shekelle P., Stewart L.A., PRISMA-P Group (2015). Preferred reporting items for systematic review and meta-analysis protocols (PRISMA-P) 2015 statement. Syst. Rev..

[12] National Health and Medical Research Council (2009). NHMRC Levels of Evidence and Grades for Recommendations for Guideline Developers.

[13] Higgins J.P., Altman D.G., Gøtzsche P.C., Jüni P., Moher D., Oxman A.D., Savovic J., Schulz K.F., Weeks L., Sterne J.A. (2011). The Cochrane Collaboration’s tool for assessing risk of bias in randomised trials. BMJ.

[14] American Dietetic Association (2008). Evidence Analysis Manual: Steps in the ADA Evidence Analysis Process.

[15] Cai W., Cijiang J., Zhu L., Peppa M., Lu C., Uribarri J., Vlassara H. (2004). High levels of dietary advanced glycation end products transform low-density lipoprotein into a potent redox-sensitive mitogen-activated protein kinase stimulant in diabetic patients. Circulation.

[16] Harcourt B.E., Sourris K.C., Coughlan M.T., Walker K.Z., Dougherty S.L., Andrikopoulos S., Morley A.L., Thallas-Bonke V., Chand V., Penfold S.A. (2011). Targeted reduction of advanced glycation improves renal function in obesity. Kidney Int..

[17] Luévano-Contreras C., Garay-Sevilla M.E., Wrobel K., Malacara J.M., Wrobel K. (2013). Dietary advanced glycation end products restriction diminishes inflammation markers and oxidative stress in patients with type 2 diabetes mellitus. J. Clin. Biochem. Nutr..

[18] Mark A.B., Poulsen M.W., Andersen S., Andersen J.M., Bak M.J., Ritz C., Holst J.J., Nielsen J., de Courten B., Dragsted L.O. (2014). Consumption of a diet low in advanced glycation end products for 4 weeks improves insulin sensitivity in overweight women. Diabetes Care.

[19] Peppa M., Uribarri J., Cai W., Lu M., Vlassara H. (2004). Glycoxidation and inflammation in renal failure patients. Am. J. Kidney Dis..

[20] Semba R.D., Gebauer S.K., Baer D.J., Sun K., Turner R., Silber H.A., Talegawkar S., Ferrucci L., Novotny J.A. (2014). Dietary intake of advanced glycation end products did not affect endothelial function and inflammation in healthy adults in a randomized controlled trial. J. Nutr..

[21] Uribarri J., Cai W., Pyzik R., Goodman S., Chen X., Zhu L., Ramdas M., Striker G.E., Vlassara H. (2014). Suppression of native defense mechanisms, SIRT1 and PPAR, by dietary glycoxidants precedes disease in adult humans; relevance to lifestyle-engendered chronic diseases. Amino Acids.

[22] Uribarri J., Cai W., Ramdas M., Goodman S., Pyzik R., Xue C., Zhu L., Striker G.E., Vlassara H. (2011). Restriction of advanced glycation end products improves insulin resistance in human type 2 diabetes: Potential role of AGER1 and SIRT1. Diabetes Care.

[23] Uribarri J., Peppa M., Cai W., Goldberg T., Lu M., Baliga S., Vassalotti J.A., Vlassara H. (2003). Dietary glycotoxins correlate with circulating advanced glycation end product levels in renal failure patients. Am. J. Kidney Dis..

[24] Vlassara H., Cai W., Crandall J., Goldberg T., Oberstein R., Dardaine V., Peppa M., Rayfield E.J. (2002). Inflammatory mediators are induced by dietary glycotoxins, a major risk factor for diabetic angiopathy. Proc. Natl. Acad. Sci. USA.

[25] Vlassara H., Cai W., Goodman S., Pyzik R., Yong A., Chen X., Zhu L., Neade T., Beeri M., Silverman J.M. (2009). Protection against loss of innate defenses in adulthood by low advanced glycation end products (AGE) intake: Role of the antiinflammatory AGE receptor-1. J. Clin. Endocrinol. Metab..

[26] Goldberg T., Cai W., Peppa M., Dardaine V., Baliga B.S., Uribarri J., Vlassara H. (2004). Advanced glycoxidation end products in commonly consumed foods. J. Am. Diet. Assoc..

[27] El Nahas A.M., Bello A.K. (2005). Chronic kidney disease: The global challenge. Lancet.

[28] Bierhaus A., Schiekofer S., Schwaninger M., Andrassy M., Humpert P.M., Chen J., Hong M., Luther T., Henle T., Klöting I. (2001). Diabetes-Associated Sustained Activation of the Transcription Factor Nuclear Factor-κB. Diabetes.

[29] Goldin A., Beckman J.A., Schmidt A.M., Creager M.A. (2006). Advanced Glycation End Products: Sparking the Development of Diabetic Vascular Injury. Circulation.

[30] Scheller J., Chalaris A., Schmidt-Arras D., Rose-John S. (2011). The pro- and anti-inflammatory properties of the cytokine interleukin-6. Biochim. Biophys. Acta.

[31] Moller D.E. (2000). Potential role of TNF-α in the pathogenesis of insulin resistance and type 2 diabetes. Trends Endocrinol. Metab..

[32] Basu S. (2008). F2-isoprostanes in human health and diseases: From molecular mechanisms to clinical implications. Antioxid. Redox Signal..

[33] Ceriello A. (2003). New insights on oxidative stress and diabetic complications may lead to a “causal” antioxidant therapy. Diabetes Care.

[34] Forbes J.M., Coughlan M.T., Cooper M.E. (2008). Oxidative stress as a major culprit in kidney disease in diabetes. Diabetes Care.

[35] Seiquer I., Rubio L.A., Peinado M.J., Delgado-Andrade C., Navarro M.P. (2014). Maillard reaction products modulate gut microbiota composition in adolescents. Mol. Nutr. Food Res..

[36] Cani P.D., Bibiloni R., Knauf C., Waget A., Neyrinck A.M., Delzenne N.M., Burcelin R. (2008). Changes in gut microbiota control metabolic endotoxemia-induced inflammation in high-fat diet—Induced obesity and diabetes in mice. Diabetes.

[37] Cani P.D., Possemiers S., Van de Wiele T., Guiot Y., Everard A., Rottier O., Geurts L., Naslain D., Neyrinck A., Lambert D.M. (2009). Changes in gut microbiota control inflammation in obese mice through a mechanism involving GLP-2-driven improvement of gut permeability. Gut.

[38] Coughlan M.T., Yap F.Y.T., Tong D.C.K., Andrikopoulos S., Gasser A., Thallas-Bonke V., Webster D.E., Miyazaki J., Kay T.W., Slattery R.M. (2011). Advanced glycation end products are direct modulators of β-Cell function. Diabetes.

[39] Hofmann S.M., Dong H.-J., Li Z., Cai W., Altomonte J., Thung S.N., Zeng F., Fisher E.A., Vlassara H. (2002). Improved insulin sensitivity is associated with restricted intake of dietary glycoxidation products in the db/db mouse. Diabetes.

[40] Sandu O., Song K., Cai W., Zheng F., Uribarri J., Vlassara H. (2005). Insulin resistance and type 2 diabetes in high-fat–fed mice are linked to high glycotoxin intake. Diabetes.

[41] Birlouez-Aragon I., Saavedra G., Tessier F.J., Galinier A., Ait-Ameur L., Lacoste F., Niamba C.N., Alt N., Somoza V., Lecerf J.M. (2010). A diet based on high-heat-treated foods promotes risk factors for diabetes mellitus and cardiovascular diseases. Am. J. Clin. Nutr..

[42] Apple F.S., Christenson R.H., Danne O., Jaffe A.S., Mair J., Mockel M., Pagani F., Christenson R.H., Mockel M., Danne O. (2005). Future biomarkers for detection of ischemia and risk stratification in acute coronary syndrome. Clin. Chem..

[43] Tsimikas S., Willeit P., Willeit J., Santer P., Mayr M., Xu Q., Mayr A., Witztum J.L., Kiechl S. (2012). Oxidation-specific biomarkers, prospective 15-year cardiovascular and stroke outcomes, and net reclassification of cardiovascular events. J. Am. Coll. Cardiol..

[44] Cerami C., Founds H., Nicholl I., Mitsuhashi T., Giordano D., Vanpatten S., Lee A., Al-Abed Y., Vlassara H., Bucala R., Cerami A. (1997). Tobacco smoke is a source of toxic reactive glycation products. Proc. Natl. Acad. Sci. USA.

[45] Pouillart P., Mauprivez H., Ait-Ameur L., Cayzeele A., Lecerf J.M., Tessier F.J., Birlouez-Aragon I. (2008). Strategy for the study of the health impact of dietary Maillard products in clinical studies: The example of the ICARE clinical study on healthy adults. Ann. N. Y. Acad. Sci..

[46] Ames J.M. (2008). Determination of Nɛ-(Carboxymethyl)lysine in foods and related systems. Ann. N. Y. Acad. Sci..

